# Giant omental lipoma, a rare etiology of right-iliac fossa pain in adult: A surgical case report

**DOI:** 10.1016/j.ijscr.2022.107428

**Published:** 2022-07-23

**Authors:** Athary Saleem, Jumana Alfadhli, Abrar Alawadhi, Maher Hassan, Khaled Alshammari

**Affiliations:** Department of General Surgery, Al-Adan Hospital, Kuwait

**Keywords:** Intraperitoneal mass, Lipoma, Omentum, Abdominal pain, Case report

## Abstract

**Background:**

Omental lipoma is an uncommon abdominal tumor of mature fat cells. Those benign tumors are usually asymptomatic but occasionally can cause signs and symptoms based on their location, size, and presence of complications. Radiological investigations such as Abdominal ultrasonography (USG) and computed tomography (CT) are crucial to evaluate and diagnose intra-abdominal tumors, especially omental lipomas.

**Presentation:**

A 61-year-old male patient presented to our hospital with right iliac fossa pain. Physical examination and laboratory test results were normal. The performed abdominal CT scan revealed a large right-sided intraperitoneal mass measuring about 2.4 × 10 × 20 cm. Then, an ultrasound-guided biopsy was done and the picture was most consistent with lipoma. So, surgical intervention was decided and omental lipoma was completely exteriorized via a laparoscopic approach. The weight of the excised omental mass was 2.45 kg, measuring 23 × 18 × 7 cm. The resected specimens, including omental lipoma and omental lymph nodes, were sent for histopathological studies. The postoperative period was uneventful.

**Discussion:**

Omental lipoma is an unusual entity that occurs often in children and rarely in adults. The clinical features of omental lipomas include abdominal discomfort, abdominal lump, abdominal pain, nausea, and/or weight loss. Diagnosis of the omental lipoma relies on imaging and physical examination, which was normal in the presented case. Abdominal CT provides definitive fat content characterization and its localization within the omentum.

**Conclusion:**

Due to the rare etiologic origin of omental lipomas, we report the case of a 61-year-old male with right iliac fossa pain, found to be caused by detected giant omental lipoma.

## Highlights

• Omental lipomas are extremely rare soft tissue tumors.
• Omental lipomas are uncommon in adults.
• The intraoperative detection of a giant omental lipoma is considered an unusual finding.
• Both CT and USG provide high diagnostic value for intraabdominal lesions.
• The laparoscopic approach is the best treatment option.

## 1 Introduction

Despite being the most common benign mesenchymal tumors of soft tissue, omental lipomas are extremely rare [1], [2], [3], [4]. In an adult, giant omental lipomas account for less than ten published cases reported worldwide [5]. Typically, patients with omental lipomas are asymptomatic, while large lipomas can cause non-specific symptoms and abdominal distension [3], [4]. Here we report a 61-year-old gentleman who presented with right iliac fossa pain. Our work has been reported in line with the SCARE 2020 criteria [6].

## 2 Case presentation

**Fig. 1:**
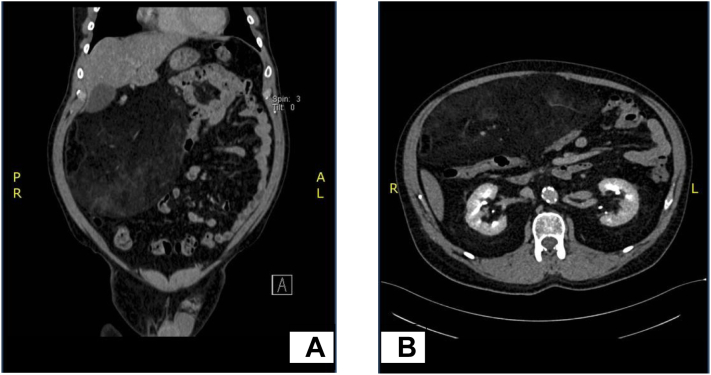
Omental lipoma case. A: Coronal abdominal CT scan demonstrating a well-defined heterogeneous lesion associated with mass effects compressing the transverse colon. B: Axial CT scan shows lobulated and encapsulated lesions.

A 61-year-old male patient, with a previous medical history of diabetes mellitus, hypertension, ischemic heart disease, and repaired paraumbilical hernia with mesh, presented to our hospital with right iliac fossa pain. Physical examination and laboratory test results were all normal. At this point, acute appendicitis was suspected and an abdominal CT scan was suggested to rule it out. An abdominal CT scan revealed a large right-sided intraperitoneal mass measuring about 2.4 × 10 × 20 cm (Fig. 1).

**Fig. 2:**
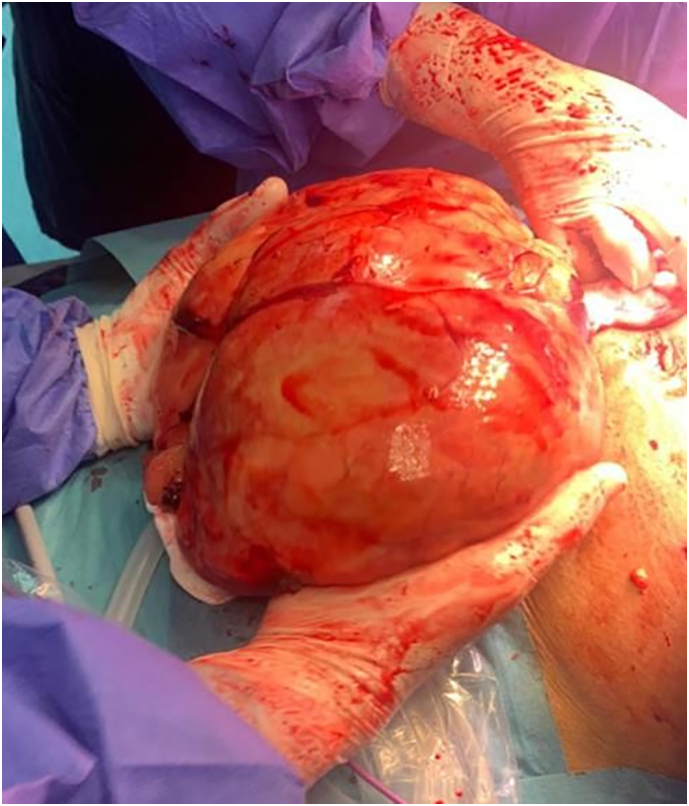
Image of the detected omental lipoma intraoperatively.

As a result, an ultrasound-guided biopsy was done. It revealed mature adipose tissue with hemorrhage and no evidence of atypia. The picture was most consistent with lipoma. So, surgical intervention was decided and laparoscopic excision of radiologically detected lipoma was scheduled. The complete dissection of the large omental mass was done laparoscopically. Then, the omental lipoma was exteriorized via an extension of the midline port side incision along with omental lymph nodes were detected during the procedure. The weight of the excised omental mass, which was detected intraoperatively, was 2.45 kg (Fig. 2).

**Fig. 3:**
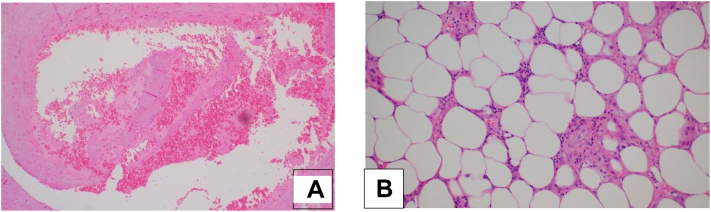
Microscopic features of the omental lipoma. A: Hemorrhage within lipoma. B: mature fat cells and fat necrosis.

Macroscopically, the omental mass consists of a soft fatty mass measuring 23 × 18 × 7 cm, showing a smooth yellowish outer surface with dark brownish hemorrhagic areas. Whereas, the omental lymph node consists of irregular gray-brown soft fragments measuring 1.5 × 1.3 × 0.6 cm. Then, the omental mass and lymph node specimens were sent for further histopathological studies (Fig. 3). The pathology showed lipomatous tumor consistent with lipoma with extensive fat necrosis with no evidence of nuclear atypia and infarcted bulk of lesion. Furthermore, a fibrovascular tissue component of the omental lymph nodes was detected. The postoperative period was uneventful, and the patient was discharged on the third postoperative day.

## 3 Discussion

Abdominal cavity lipomas can present in the mesentery, omentum, and retroperitoneum, but the omental lipomas are rare [7]. Omental lipoma is an unusual entity that occurs often in children and rarely in adults [1], [2]. The reported cases of omental lipomas are limited in the literature [3], [5]. Lipomas can be single or multiple, superficial or deeply localized [8], [9]. Usually, omental lipomas are asymptomatic [2], [7], [10]. The clinical presentation varies depending on the location, size, and presence of complications [3], [11]. The clinical features of omental lipomas include abdominal discomfort, abdominal lump, abdominal pain, nausea, and/or weight loss [1], [8], [11]. Large-sized lipomas may lead to bowel obstructions and distension [9]. Initially, the omental lipoma diagnosis relies on physical examination that includes mass inspection and palpation [4], [5], [12]. In the presented case, the physical examination was normal. Abdominal ultrasonography (USG) and computed tomography (CT) play an important role in the evaluation and diagnosis of intra-abdominal tumors [1], [5], [11], [12]. On USG, the intra-abdominal lipomas can be detected as homogeneous echogenic masses with irregular borderlines. Furthermore, an abdominal CT scan adds a greater diagnostic value of omental lipomas [11], [12]. It provides definitive fat content characterization by determining the attenuation values (−80 to −120 HU) that leads to the lesion localization within the omentum [4]. Although USG and CT are important diagnostic tools, MRI adds better demonstration of the surrounding anatomy [10]. Both CT and MRI help in the detection of complications, such as bleeding or torsion, that may develop in symptomatic patients [1], [4]. In our case, an abdominal CT scan was performed, revealing a right-sided large intra-abdominal mass-like lesion described as:

• Large well-defined mixed hypo and hyperdense misty soft-tissue attenuation lesion
• The lesion is seen on the right hypochondria and extends to the midline of the abdomen
• It measures about 2.4 × 10 × 20 cm
• Lesion associated with mass effect compressing the transverse colon inferiorly
• No radiological evidence of acute appendicitis

The definitive treatment modality of omental lipoma is surgical resection [1], [7]. Surgical excision can be achieved by the laparoscopic technique [1], [5]. The preferred surgical method depends on the mass size and localization, its surrounding tissues, and patient-related factors [1], [8]. Complete resection of the omental lipomas is crucial to avoid recurrence. After excision, the recurrence rate is less than 5 % [5], [13]. In the current case, the omental lipoma was excised with its omental lymph nodes through a laparoscopic technique and delivered via a midline incision.

## 4 Conclusion

Despite the rarity of omental lipomas, they should be considered in unusual abdominal pain or vague non-specific symptoms. Imaging helps to demonstrate the characteristics and localization of the intraabdominal lipomas. To cure omental lipoma, total excision via a laparoscopic approach is a viable treatment option. Our case report emphasizes the diagnostic and surgical challenges of omental lipomas.

## Ethical approval

This report does not contain any personal information that could lead to the identification of the patient.

## Provenance and peer review

Not commissioned, externally peer-reviewed.

## Sources of funding

No sources of funding are available.

## Consent

Written informed consent was obtained from the patient for publication of this case report and accompanying images. A copy of the written consent is available by the Editor-in-Chief of this journal upon request.

## Author contribution

AS wrote and edited the manuscript; KA, MH, and AA operate; AS and KA provided the illustrated figures; JA and AA analyzed the data; all authors read, contribute, and approved the final manuscript.

## Research registration

This does not apply to our case report, there is no involvement of research here.

## Guarantor

Athary Saleem, B.Med.Sc., MD; Department of General Surgery, Al Adan Hospital, Kuwait.

## Declaration of competing interest

There is no conflict of interest including any financial or personal relationships with other people or organizations or any work influencers.
